# The multifaceted phenotypic and genotypic spectrum of type-IV-collagen-related nephropathy—A human genetics department experience

**DOI:** 10.3389/fmed.2022.957733

**Published:** 2022-08-31

**Authors:** Jasmina Ćomić, Korbinian M. Riedhammer, Roman Günthner, Christian W. Schaaf, Patrick Richthammer, Hannes Simmendinger, Donald Kieffer, Riccardo Berutti, Velibor Tasic, Nora Abazi-Emini, Valbona Nushi-Stavileci, Jovana Putnik, Nataša Stajic, Adrian Lungu, Oliver Gross, Lutz Renders, Uwe Heemann, Matthias C. Braunisch, Thomas Meitinger, Julia Hoefele

**Affiliations:** ^1^Institute of Human Genetics, Klinikum rechts der Isar, Technical University of Munich, School of Medicine, Munich, Germany; ^2^Department of Nephrology, Klinikum rechts der Isar, Technical University of Munich, School of Medicine, Munich, Germany; ^3^University Children's Hospital, Medical Faculty of Skopje, Skopje, North Macedonia; ^4^Pediatric Clinic, University Clinical Center of Kosovo, Prishtina, Serbia; ^5^Institute for Mother and Child Health Care of Serbia “Dr. Vukan Čupić”, Department of Nephrology, University of Belgrade, Faculty of Medicine, Belgrade, Serbia; ^6^Clinic of Nephrology and Rheumatology, University Medical Center Göttingen, University of Göttingen, Göttingen, Germany

**Keywords:** type-IV-collagen-related nephropathy, Alport syndrome, *COL4A3*, *COL4A4*, *COL4A5*

## Abstract

Disease-causing variants in *COL4A3-*5 are associated with type-IV-collagen-related nephropathy, a genetically and phenotypically multifaceted disorder comprising Alport syndrome (AS) and thin basement membrane nephropathy (TBMN) and autosomal, X-linked and a proposed digenic inheritance. Initial symptoms of individuals with AS are microscopic hematuria followed by proteinuria leading to kidney failure (90% on dialysis < age 40 years). In contrast, individuals with TBMN, an outdated histology-derived term, present with microscopic hematuria, only some of them develop kidney failure (>50 years of age). An early diagnosis of type-IV-collagen-related nephropathy is essential for optimized therapy and slowing of the disease. Sixty index cases, in whom exome sequencing had been performed and with disease-causing variant(s) in *COL4A3-5*, were evaluated concerning their clinical tentative diagnosis and their genotype. Of 60 reevaluated individuals with type-IV-collagen-related nephropathy, 72% had AS, 23% TBMN and 5% focal segmental glomerulosclerosis (FSGS) as clinical tentative diagnosis. The FSGS cases had to be re-classified as having type-IV-collagen-related nephropathy. Twelve percent of cases had AS as clinical tentative diagnosis and a monoallelic disease-causing variant in *COL4A3/4* but could not be classified as autosomal dominant AS because of limited or conflicting clinical data. This study illustrates the complex clinical and genetic picture of individuals with a type IV-collagen-related nephropathy indicating the need of a refined nomenclature and the more interdisciplinary teamwork of clinicians and geneticists as the key to optimized patient care.

## Introduction

The α3, α4, and α5 chains of the type IV collagen are an essential component of the glomerular basement membrane (GBM) and are encoded by the three genes *COL4A3, COL4A4*, and *COL4A5* (1). Disease-causing variants [(likely) pathogenic and pathogenic variants as per the guideline for sequence variant interpretation of the American College of Medical Genetics and Genomics and current amendments; see Material and Methods] in one of these genes are associated with type-IV-collagen-related nephropathy, comprising Alport syndrome (AS) and thin basement membrane nephropathy (TBMN) (2–8).

AS is characterized by microscopic hematuria and proteinuria leading to progressive loss of kidney function. Additionally, sensorineural hearing impairment, and eye abnormalities can be observed. AS is the second most common monogenic cause for kidney failure (1). It can be inherited in an X-linked [XLAS; hemizygous (male) or heterozygous (female) disease-causing variant in *COL4A5*] or autosomal recessive (ARAS; biallelic pathogenic variants in *COL4A3*/*COL4A4*) form (1, 9–11). The often used designation autosomal dominant AS in carriers of monoallelic pathogenic variants in *COL4A3* and *COL4A4* is differently used in the literature. In one recent publication by Furlano et al., the authors propose that any case harboring one heterozygous disease-causing variant in *COL4A3* or *COL4A4* should be designated as autosomal dominant AS independently from the clinical phenotype which ranges from microscopic hematuria to chronic kidney disease (12). In contrast, Savige et al. classify individuals with a heterozygous disease-causing variant in *COL4A3* or *COL4A4* as having autosomal dominant inherited TBMN or AS depending on the clinical phenotype and a potential positive familial history (13, 14). Furthermore, digenic inheritance has also been discussed as a possible cause in individuals with AS (15–18).

TBMN is a histopathology-derived term defined as uniform thinning of the GBM and phenotypically characterized by persistent microscopic hematuria, minimal if any proteinuria, and normal renal function (19, 20). Solely thinning of the GBM can also be found in early stages of AS (19, 21). The frequency of TBMN has been estimated to be as high as 1% of the world population (22). In up to 20% of the individuals with TBMN, disease progression to late-onset—compared to AS—kidney failure (>50 years of age) has been reported (23). This disease progress seems to be related in part to the development of focal segmental glomerulosclerosis (FSGS) (9, 24). Hence, in some cases with suspicion of a hereditary podocytopathy (hereditary FSGS), disease-causing variants in *COL4A3*-*COL4A5* can be found (25, 26).

The focus of this study was the reevaluation of the clinical phenotype and the reanalysis of exome sequencing data of 60 individuals with disease-causing variants in *COL4A3-5* in order to evaluate and highlight the shortcomings of the current nomenclature of AS/TBMN.

## Methods

### Study population

For this study, a cohort of 60 index cases of unrelated families with disease-causing variants in *COL4A3, COL4A4*, or *COL4A5* was investigated. These families have been recruited between October 2015 and August 2020 according to their appearance at our institute. In all individuals exome sequencing was already performed and genetic data were available. The exome data were reanalyzed in *COL4A3-5* within this study. This study was carried out according to standards of the 2013 Helsinki Declaration and authorized by the local Ethics Committee of the Technical University of Munich. Informed and written consents were obtained from all individuals or their legal guardians.

### Clinical case information

Clinical and phenotypic information were obtained from clinical reports and medical history. Additionally, a standardized questionnaire was used to evaluate clinical information. The individuals were assigned to one of the following groups according to the clinical tentative diagnoses/kidney biopsy results as assigned by the referring clinician (nephrologists or pediatric nephrologists): AS, TBMN or FSGS. Age of onset of kidney failure in individuals was determined as the beginning of renal replacement therapy (hemodialysis or peritoneal dialysis) or pre-emptive kidney transplantation.

### Genetic testing

For extraction of DNA from peripheral blood the automated nucleic acid purification instrument Chemagic™ 360 (PerkinElmer, Waltham, MA, USA) according to the manufacturer's protocol was used.

### Exome sequencing

Exome sequencing was performed with Sure Select Human All Exon 60Mb V6 Kit (Agilent) and on a HiSeq4000 platform (Illumina) in the index cases (27). Mitochondrial DNA was derived from off-target exome reads as previously described (28). Reads were aligned to the human reference genome (UCSC Genome Browser build hg19) using Burrows-Wheeler Aligner (v.0.7.5a). Using SAMtools (version 0.1.19), detection of single-nucleotide variants (SNVs) and small insertions and deletions (indels) was accomplished. For investigation of copy number variants (CNVs) (including exon-spanning intronic regions) ExomeDepth was used. A noise threshold of 2.5 was accepted (29). The called CNVs were visualized by the Integrative Genomics Viewer (IGV, https://software.broadinstitute.org/software/igv/) to check if there was enough coverage of the examined regions and for plausibility of the CNVs. CNVs were then compared with publicly available control databases like the Genome Aggregation Database (gnomAD, https://gnomad.broadinstitute.org/about), the Database of Genomic Variants (DGV, http://dgv.tcag.ca/dgv/app/home) the databases for pathogenic CNVs like DECIPHER (https://decipher.sanger.ac.uk/) and ClinVar (https://www.ncbi.nlm.nih.gov/clinvar/). For the analysis of *de novo*, autosomal dominant and mitochondrial SNVs and indels, only variants with a minor allele frequency (MAF) of <0.1% (Munich Exome Server with over 22,000 exomes) were considered. For the analysis of autosomal recessive and X-linked SNVs and indels [homozygous, hemizygous or (putatively) compound heterozygous], only variants with a MAF of <1.0% were considered.

### Sanger sequencing

Using Sanger sequencing, segregation analysis was conducted. Oligonucleotide primer sequences are available upon request.

### Variant interpretation

Publicly available databases for (likely) pathogenic variants were used for comparison of all variants found and described in this study. These databases are ClinVar, the Human Gene Mutation Database (HGMD^®^, http://www.hgmd.cf.ac.uk), and the Leiden Open Variation Database (LOVD, https://www.lovd.nl). The variants were rated in accordance to American College of medical Genetics and Genomics (ACMG) guidelines and current amendments (4–7). Likely pathogenic and pathogenic variants are summarized as “disease-causing variant” in the text.

Biallelic disease-causing variants in *COL4A3* and *COL4A4* in male and female individuals, a hemizygous disease-causing variant in *COL4A5* in a male individual and a heterozygous disease-causing variant in *COL4A5* in a female individual were in accordance with the clinical tentative diagnosis of AS. In females with the clinical tentative diagnosis of TBMN and a heterozygous disease-causing variant in *COL4A5*, the genotype was also fitting to the clinical tentative diagnosis, as females with heterozygous disease-causing variants in *COL4A5* can show a broad phenotypic spectrum ranging from TBMN to AS. In contrast to that, individuals with a heterozygous (likely) pathogenic variant in *COL4A3* or *COL4A4* and a clinical tentative diagnosis of AS were not automatically seen in accordance with autosomal dominant AS but further pedigree and phenotypic information were scrutinized (see Results). In turn, if cases had the clinical tentative diagnosis of TBMN and carried a heterozygous (likely) pathogenic variant in *COL4A3* or *COL4A4* (female and male individuals), genotype and phenotype were in agreement. Carriers of a hemizygous (likely) pathogenic variant in *COL4A5* and the clinical tentative diagnosis of TBMN were classified as genetically solved AS due to the unquestionable genotype of hemizygous disease-causing variants in *COL4A5* leading to AS (which can be mistaken as TBMN in early stages of disease). Furthermore, individuals with the non-specific phenotype of FSGS on kidney biopsy and disease-causing variants in *COL4A3, COL4A4*, or *COL4A5* were reclassified as type-IV-collagen-nephropathies (two cases ARAS, one case XLAS—female carrier; see Supplementary Table 1).

## Results

### Study population

**Figure 1:**
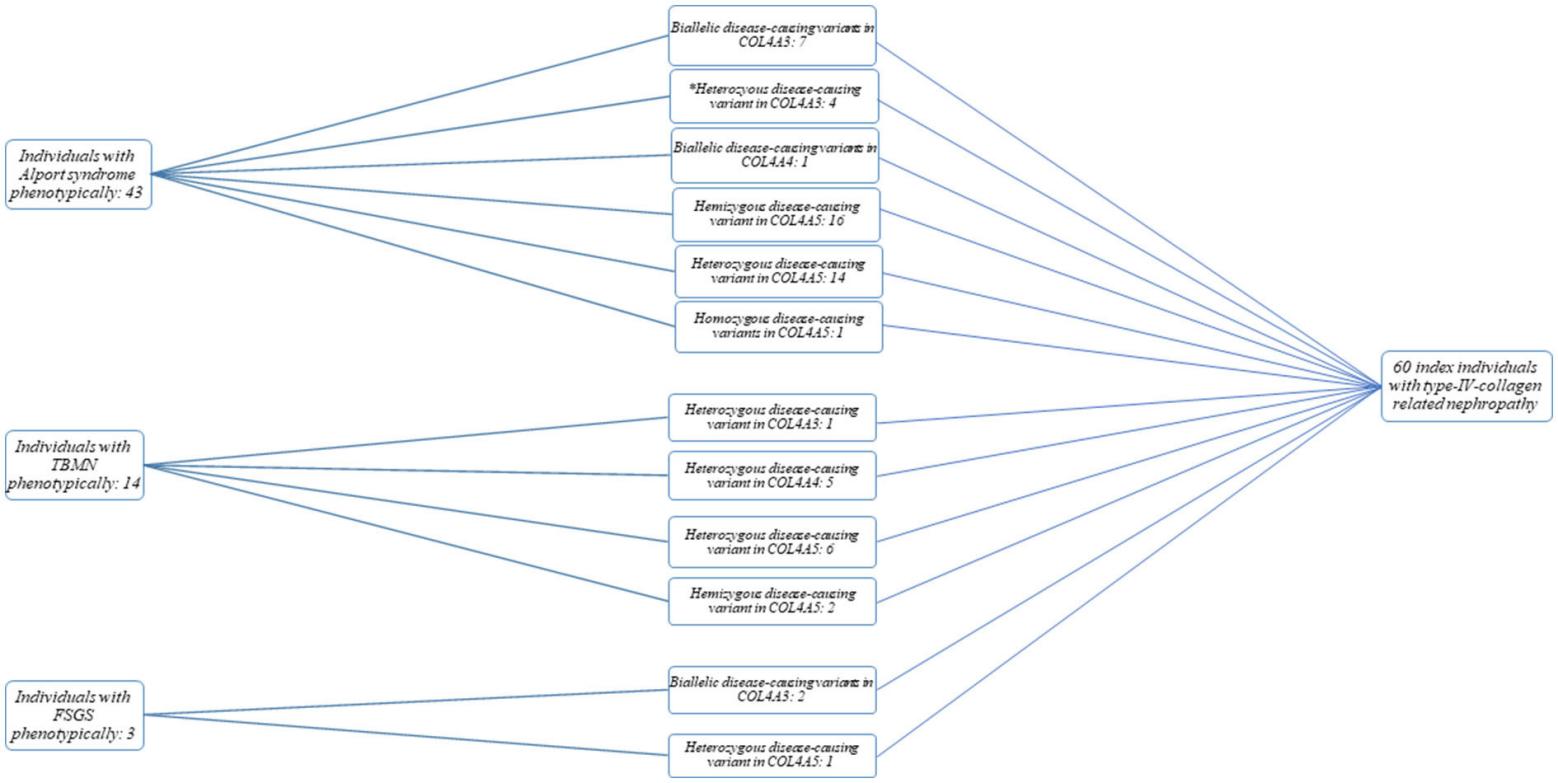
Flow chart and cohort overview. Categorization of individuals into Alport syndrome (AS), thin basement membrane nephropathy (TBMN), and focal segmental glomerulosclerosis (FSGS) phenotypic subgroups was based on clinical tentative diagnosis/kidney biopsy result. *, Individuals with a questionable phenotype.

The total study cohort consisted of 60 unrelated index cases (30 females and 30 males) with disease-causing variants in *COL4A3-5*. 43/60 (72%) individuals had AS phenotypically, 14/60 (23%) TBMN and 3/60 (5%) FSGS on biopsy (Figure 1). 55/60 individuals (92%) were of non-Finnish European descent. The median age of disease-onset was 7 years (range: 0–35 years of age). In nine cases, the age of disease-onset was not available. Clinical findings were as follows (cases with reported phenotype information): 25/25 (100%) individuals presented with microscopic hematuria (no data available from 35 individuals), 12/25 (48%) with the clinical phenotype of AS, 11/25 (44%) with TBMN, and 2/25 (8%) with FSGS. Proteinuria was seen in 15/25 (60%) individuals [no data available from 45 individuals; 10/15 (67%) with AS, 3/15 (20%) with TBMN, 2/15 (13%) with FSGS]. End-stage kidney failure (ESKF) was seen in 3/17 (18%) individuals, 2/3 (67%) with AS (23 and 24 years of age), and 1/3 (33%) with TBMN (68 years of age). No data were available from 43 individuals. Eye anomalies could be observed in 8/53 (15%) individuals (no data available from 7 individuals); all of them had AS as clinical tentative diagnosis. 18/53 (34%) individuals had hearing impairment (no data available from 7 individuals), 16/18 (89%) had AS, 2/18 (11%) TBMN.

### Coverage of genes *COL4A3-5*

The median coverage > 20x of all exons of the three genes *COL4A3-5* was > 95% (range: 88–100%; see Table 1). All exons were covered at least 10x.

**Table 1:** Exon coverage of genes *COL4A3-5* in exome sequencing.

**Gene**	**Chromosomal location**	**Transcript number**	**Inheritance**	**Phenotype MIM number**	**Covered exons (>20x)**
*COL4A3*	2q36.3	NM_000091.4	AD, AR	104200, 203780	98%
*COL4A4*	2q36.3	NM_000092.4	AD, AR	104200, 203780	100%
*COL4A5*	Xq22.3	NM_033380.3	XL	301050	88%

*AD, autosomal dominant; AR, autosomal recessive; AS, Alport syndrome; XL, X-linked*.

### Identification of different disease-causing variants

Within this study, 69 disease-causing variants could be identified, 61/69 (88%) of them were different. 26/61 (43%) were already described in the literature (*COL4A3*: 11, *COL4A4*: 1, *COL4A5*: 14), 35/61 (57%) were novel (i.e., not previously reported as disease-causing) (*COL4A3*: 9, *COL4A4*: 5, *COL4A5*: 21) (Supplementary Tables 1, 2).

### Distribution of identified (likely) pathogenic variants in *COL4A3-5*

From the 43 individuals with AS phenotypically, 39 (91%) cases had either an autosomal recessive (biallelic disease-causing variants in *COL4A3* or *COL4A4*) or X-linked AS [hemizygous (male individuals) or heterozygous/homozygous (female individuals) variants in *COL4A5*] (Figure 1; Supplementary Table 1). Of these 39 cases, 7 (18%) had (likely) pathogenic compound heterozygous/homozygous variants in *COL4A3*, 1 (3%) had a likely pathogenic homozygous variant in *COL4A4*, and 31 (79%) had a heterozygous (females) (14/31), homozygous (female; 1/31) or hemizygous (16/31) (likely) pathogenic variant in *COL4A5*.

Of the 14 individuals with TBMN phenotypically, one (7%) case had a (likely) pathogenic heterozygous variant in *COL4A3*, five (36%) in *COL4A4* and 8 (57%) in *COL4A5* (hemizygous variant: 2; heterozygous variant: 6).

Two individuals with the histopathological picture of FSGS had compound heterozygous likely pathogenic variants in *COL4A3*, and one female individual carried a heterozygous likely pathogenic variant in *COL4A5* (Supplementary Table 1).

**Figure 2:**
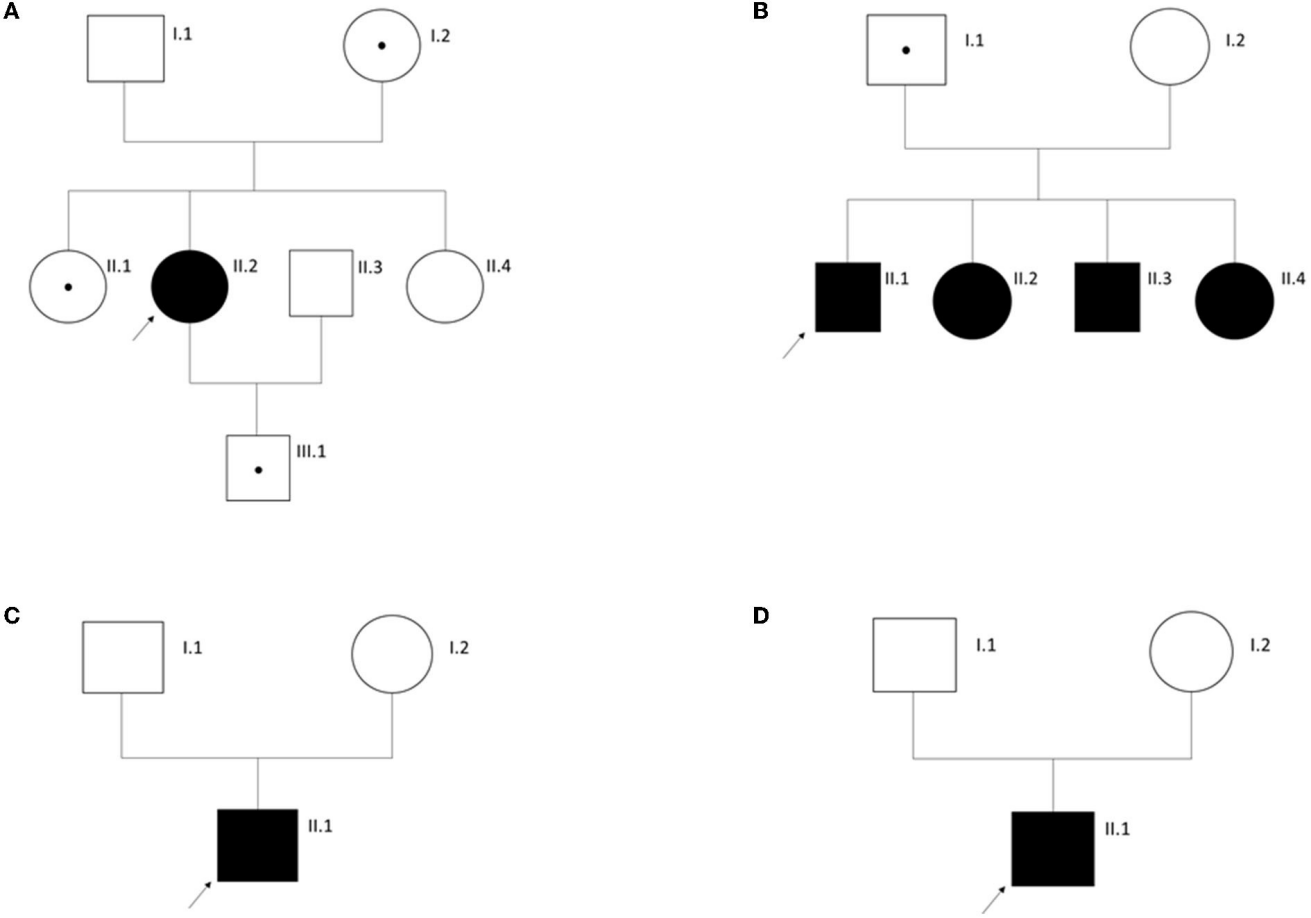
Pedigrees of families with Alport syndrome as clinical tentative diagnosis in the index individual (arrow) and only a monoallelic disease-causing variant in *COL4A3*. See Results for more information. **(A)** Family ATS-F521; **(B)** Family ATS-F663; **(C)** Family ATS-F787; **(D)** Family ATS-F788. Circles, females. Squares, males. A dot within a symbol signifies an unaffected carrier of the respective disease-causing variant.

In 4 (12%) individuals with AS phenotypically, only one heterozygous (likely) pathogenic variant in *COL4A3* gene could be identified (Supplementary Table 1). A genetic diagnosis of autosomal dominant AS was not made in these individuals because of limited or conflicting clinical data questioning their submitted diagnosis of AS. The healthy mother (62 years of age), one healthy sister (33 years of age), and the healthy son of ATS-F521-II-2 (16 years of age) also carry the variant in *COL4A3*. One additional sister of ATS-F521-II-2 also suffers from hearing impairment but has no renal phenotype. She does not carry the variant in *COL4A3* (Figure 2A). ATS-F663-II-1 inherited the variant from his healthy father but has three further affected siblings who all carry the variant. Two of the altogether four siblings additionally carry a heterozygous maternally inherited likely pathogenic variant in *MYH9* (NM_002473.4; c.1960C>G, p.(Leu654Val)). To our knowledge, the mother is healthy (Figure 2B). ATS-F787-II-1 was submitted as having AS confirmed by kidney biopsy (original biopsy report not available). No clinical data or medical records of the index and affected family members could be gathered (Figure 2C). Clinical data of ATS-F788-II-1 were also not available and a kidney biopsy confirming the clinical diagnosis of AS was not performed. This individual was part of the EARLY PRO-TECT Alport trial and medicated with placebo. Under this treatment no disease progress was seen during the trial (30) (Figure 2D).

None of the 60 individuals with disease-causing variants in one of the *COL4A3*-5 genes had additional variants in one of the two alternate *COL4A3-5* genes (Figure 1).

### Reclassification of individuals concerning their genetic result

Out of 56 individuals with a distinct genotype, the three individuals (5%) with FSGS were treated with cyclosporin A and mycophenolate mofetil. These individuals had to be re-classified as having a type-IV-collagen-related nephropathy questioning further extensive treatment with immunosuppressive drugs.

## Discussion

Type-IV-collagen-related nephropathy is a phenotypically and genetically multifaceted disorder. It comprises various phenotypes like classic AS, a slower-progressive phenotype originally described as TBMN and unspecific biopsy phenotypes like FSGS. To add to this complexity, it features both autosomal and X-linked inheritance.

We sought to illustrate this complex phenotypic and genotypic picture: In 91% of the individuals with AS phenotypically and in 100% of the individuals with TBMN phenotypically, disease-causing variants could be identified explaining the clinical tentative diagnosis. This high detection rate might be caused by intensive clinical evaluation of the affected individuals and detailed family medical history. In 5% of the individuals with disease-causing variants in *COL4A3-5*, a clinical phenotype of FSGS could be observed. This finding was already described by Malone et al. in 2014 and others (31). In their study, 10% of individuals with single or compound heterozygous disease-causing variants in *COL4A3* or *COL4A4* had the clinical tentative diagnosis of hereditary FSGS. The main cause of this clinical and genetic difference may be the fact that FSGS is an unspecific histologic phenotype seen in the process of different kidney diseases.

Digenic inheritance is also proposed as a possible cause for type-IV-collagen-related nephropathy including AS and TBMN, although little evidence is available on this topic so far (12, 16, 18). In contrast to studies describing this inheritance pattern, we did not observe findings of this pattern of inheritance within this study. This might be due to the facts that the present study had a small cohort size, the affected individuals were of different origin compared to individuals described in the literature and was performed with different sequencing techniques. Of note, it cannot be excluded that a variant was not detected if it was located in one of the limited covered exons which mostly affects the exons 5, 11, 14, 23, 38, 41, and 48 of *COL4A5*.

Importantly, in 12% of individuals with AS as clinical tentative diagnosis and monoallelic disease-causing variants in *COL4A3*/*COL4A4*, a clear statement on autosomal dominant AS could not be made taking into account the genetic result and clinical data. There is an unresolved conflict concerning autosomal dominant AS and the frontlines seem to run across two standpoints: A “clinician-centered” view stating that clear diagnoses are important for surveillance and early treatment (12, 32); and a “geneticist-centered” view that AS is a monogenic disease with complete penetrance and progressive kidney failure (90% on dialysis by age 40 years in X-linked AS; comparable for autosomal recessive AS) (11, 14). For us and others on the genetics-side (13), there are many questions concerning the simple usage of autosomal dominant AS in any case with a monoallelic disease-causing variant in *COL4A3* and *COL4A4*: In cases with a clear AS phenotype (for example on kidney biopsy, ATS-F787-II-1 above) but only a monoallelic variant in *COL4A3*/*COL4A4*, could there be another variant on the other allele missed by routine genetic testing (e.g., intronic variant leading to a splicing defect, complex rearrangement missed by short-read-based NGS)? Should we use kidney biopsy specimen (or urine-derived renal cells) in these cases to run transcriptomics on to determine if there is a splicing defect on the other allele? Should we engage further in elucidation of rearrangements with chromosomal microarray/multiplex ligation-dependent probe amplification (MLPA) or even genome sequencing? Does the same variant cause the identical disorder, AS, in a homozygous and heterozygous state? In heterozygous carriers with monoallelic missense variants in *COL4A3*/*COL4A4*, might a dominant-negative effect come into play and explain a complete AS phenotype with high penetrance? Have phenocopies been taken into account? Has the pedigree been thoroughly investigated and relatives been tested? What if healthy parents and relatives carry the variant (as in cases ATS-F521-II-2, ATS-F663-II-1), how do we counsel these parents if they get another child? Do they have a 50% recurrence risk of AS, as in other clearly autosomal dominant diseases? And does the affected individual have a 50% risk of offspring with AS if he or she gets children? To what extent is there incomplete penetrance? The estimated prevalence of heterozygous disease-causing variants in *COL4A3*/*COL4A4* is 1 in 106, as has recently been shown (8). Are we to diagnose all of these people with autosomal dominant AS? All these questions are not satisfactorily addressed so far in the literature and need to be solved to optimize the medical care and genetic counseling of these individuals.

Undoubtedly, heterozygous carriers of disease-causing variants in *COL4A3, COL4A4*, and *COL4A5* have a higher risk of end-stage kidney failure than the general population (12, 24). They need surveillance and treatment with ACE inhibition once proteinuria/albuminuria develops (33, 34). But if we classify every of these cases as having autosomal dominant AS, we are risking not making the considerations mentioned above impeding correct diagnoses and risk calculations. Hence, a unifying gene-centered nomenclature like type-IV-collagen-related nephropathy could steer free of this conflict (35), especially as TBMN is also an outdated term which is based on histologic findings not always present or preceding pathognomonic AS changes (12) (as seen by the fact that there are two cases with a hemizygous disease-causing variant in *COL4A5*, i.e., XLAS, but rated as TBMN by the referring clinician). We still used the term “TBMN” in lack of proper alternatives and as it was used by referring clinicians. In our opinion, genetic reports should state the genotype in the diagnosis and the designation AS should only be added if there is genotypic and phenotypic evidence for this diagnosis (e.g., “Diagnosis of type-IV-collagen-related nephropathy—X-linked Alport syndrome—hemizygous pathogenic frameshift variant in *COL4A5*”).

There is lacking phenotype information (see Section Results) on a number of individuals in this study, which can be viewed as a limitation. However, the aim of this study was to illustrate the phenotypic and genotypic spectrum of type-IV-collagen related nephropathy and not a detailed genotype-phenotype correlation on AS, which have been published extensively (9–12, 36).

Finally, in this study, 57% of the identified variants were novel indicating that there are still many disease-causing variants in type-IV-collagen-releated-nephropathy unknown so far. Therefore, it is extremely important to submit identified variants to open genetic databases like ClinVar or LOVD to extend the knowledge of disease-causing variants and to optimize the clinical care of individuals with a type-IV-collagen-related nephropathy.

To conclude, this study illustrates the complex clinical and genetic spectrum of type-IV-collagen-related nephropathy including AS and TBMN in a small single tertiary-care center cohort. A refined nomenclature not impeding swift diagnosis, surveillance and treatment but owing to the diverse genetic considerations of this multifaceted disorder is direly needed and, by using the term “type-IV-collagen-related nephropathy”, we propose a more gene-centered approach. Additionally, close cooperation of clinicians and geneticists is key to collect the necessary phenotypic and pedigree data needed to adequately assess individuals with suspected type-IV-collagen-related nephropathy.

## Data availability statement

The datasets presented in this study can be found in online repositories. The names of the repository/repositories and accession number(s) can be found in the article/Supplementary material.

## Ethics statement

The studies involving human participants were reviewed and approved by Ethics Committee, Klinikum rechts der Isar, Technical University of Munich, Munich, Germany. Written informed consent to participate in this study was provided by the participants' legal guardian/next of kin. Written informed consent was obtained from the individual(s), and minor(s)' legal guardian/next of kin, for the publication of any potentially identifiable images or data included in this article.

## Author contributions

Research and study design: KMR and JH. Data analysis/interpretation: JĆ, KMR, TM, and JH. Statistical analysis: JĆ. Patient acquisition: RG, PR, HS, DK, VT, NA-E, VN-S, JP, NS, AL, LR, UH, MB, CS, TM, and JH. Drafting and revising the article: JĆ, KMR, CS, TM, and JH. Supervision or mentorship and final approval of the version to be published: JH. All author contributed important intellectual content during manuscript drafting or revision, agrees to be personally accountable for the individual's own contributions, to ensure that questions pertaining to the accuracy or integrity of any portion of the work, even one in which the author was not directly involved, are appropriately investigated and resolved, and including with documentation in the literature if appropriate.

## Funding

This work was supported with a research Grant by the European Society for Pediatric Nephrology (ESPN e.V.; ESPN #2.2020) and by the German Research Foundation (DFG) and the Technical University of Munich (TUM) in the framework of the Open Access Publishing Program.

## Conflict of interest

The authors declare that the research was conducted in the absence of any commercial or financial relationships that could be construed as a potential conflict of interest.

## Publisher's note

All claims expressed in this article are solely those of the authors and do not necessarily represent those of their affiliated organizations, or those of the publisher, the editors and the reviewers. Any product that may be evaluated in this article, or claim that may be made by its manufacturer, is not guaranteed or endorsed by the publisher.
