# Supplementary material for: The multifaceted phenotypic and genotypic spectrum of type-IV-collagen-related nephropathy—A human genetics department experience
Source: Front Med (Lausanne). 2022 Aug 31;9:957733. doi: 10.3389/fmed.2022.957733 (PMC9470833; doi:10.3389/fmed.2022.957733)
Supplement: Supplementary file 1 [file Data_Sheet_1.PDF]

The multifaceted phenotypic and genotypic spectrum of type-IV-collagen-related nephropathy – a human genetics department experience

Supplementary Tables and Legends

Supplementary Table 1	2
Supplementary Table 2	7
Supplementary Table Legends	8
References	9

Supplementary Table 1

Index Case (sex)	Origin	Family history	Consanguinity	Clinical Diagnosis (referring clinician)	Y	Microscopic Hematuria	Proteinuria	Eye anomalies	Hearing impairment	Biopsy	ESKF or preemptive kidney transplantation (age)	Gene	Chromosomal position (hg19)	Nucleotide change	Amino acid change	Zygosity	Inheritance	gnomAD v.2.1.1 MAF/occurrence (CNVs)	Genetic diagnosis	ACMG criteria and rating	Accession number in ClinVar	Individual ID in LOVD	Reference
**ATS-F6-II-2 (F)**	Germany	Yes	No	AS	16	Yes	Yes	No	No	No	No	*COL4A3*	chr2:g.228128633G>A	c.1288G>A	p.(Gly430Arg)	Heterozygous	Father	Not listed	ARAS	PM1_strong, PM2, PP3 **Likely pathogenic**	SCV001150051.1	-	Novel
												COL4A3	chr2:g.228176554C>T	c.4981C>T	p.(Arg1661Cys)	Heterozygous	Mother	0.0003596 (1homozygous)		PS4_moderate PM1, PM2, PP3 **Likely pathogenic**	SCV001149721.1	-	Heidet et al. 2001
**ATS-F9-II-1 (M)**	Germany	No	No	FSGS	29	Yes	Yes	No	No	Yes (FSGS)	No	*COL4A3*	chr2:g.228144508G>C	c.2126-1G>C	p.(?)	Heterozygous	Mother	Not listed	ARAS	PVS1, PM2 **Likely pathogenic**	SCV001430094.1	-	Novel
												COL4A3	chr2:g.228172594T>C	c.4421T>C	p.(Leu1474Pro)	Heterozygous	Father	0.002664		PS4_moderate (in trans with pathogenic variants), PM1, PM3, PP3 **Likely pathogenic**	SCV001149720.2	-	Chatterjee et al. 2013
**ATS-F29-III-1 (F)**	Germany	No	No	FSGS	12	Yes	Yes	No	No	Yes (FSGS)	No	*COL4A3*	chr2:g.228137737G>A	c.1831G>A	p.(Gly611Arg)	Heterozygous	Father	Not listed	ARAS	PS4_supporting, PM1_strong PM2, PP3 **Likely pathogenic**	SCV001150053.1	-	Novel
												COL4A3	chr2:g.228172594T>C	c.4421T>C	p.(Leu1474Pro)	Heterozygous	Mother	0.002664		PS4_moderate (in trans with pathogenic variants), PM1, PM3, PP3 **Likely pathogenic**	SCV001149720.2	-	Chatterjee et al. 2013
**ATS-F70-II-1 (F)**	Germany	Yes	No	AS	8	Yes	Yes	No	Yes	Yes (AS)	No	*COL4A3*	chr2:g.228142165G>A	c.2021G>A	p.(Gly674Asp)	Heterozygous	Mother	Not listed	ARAS	PM1_strong PM2, PM3, PP3 **Likely pathogenic**	SCV001162799.1	-	Novel
												COL4A3	chr2:g.(228163529_228167753)_(228169800_228172425)dup	Duplication of exons 44-47	-	Heterozygous	Father	Not listed		PVS1, PS4_supporting, PM2 **Pathogenic**	-	00375231	Morinière et al. 2014
**ATS-F48-II-1 (M)**	United Arab Emirates	N.A	Yes	AS	19	Yes	Yes	No	No	Yes (FSGS and FGGS)	No	*COL4A3*	chr2:g.228128528G>A	c.1183G>A	p.(Gly395Arg)	Homozygous	Unknown	Not listed	ARAS	PM1_strong, PM2, PP3 **Likely pathogenic**	SCV001150050.1	-	Novel
**ATS-F251-II-3 (M)**	Macedonia	N.A	N.A	AS	n.d.	Yes	Yes	Yes	Yes	No	No	*COL4A3*	chr2:g.228118877dup	c.816dup	p.(Pro273Serfs*14)	Heterozygous	*De-novo*	Not listed	ARAS	PVS1, PM2 **Likely pathogenic**	SCV001150049.1	-	Novel
												COL4A3	chr2:g.228145646dup	c.2417dup	p.(Gly807Argfs*28)	Heterozygous	Mother	0.000005782		PVS1, PM2 **Likely pathogenic**	SCV001150054.1	-	Heidet et al. 2001
**ATS-F259-II-1 (M)**	Serbia	Yes	No	AS	9	Yes	Yes	No	Yes	Yes (FSGS)	No	*COL4A3*	chr2:g.228155501C>T	c.3109C>T	p.(Arg1037*)	Heterozygous	Father	0.000008017	ARAS	PVS1, PS4_moderate, PM2 **Pathogenic**	SCV001149718.1	-	Longo et al. 2002
												COL4A3	chr2:g.228159715G>C	c.3454G>C	p.(Gly1152Arg)	Heterozygous	Mother	Not listed		PS4_supporting, PM1_strong, PM2, PM3, PP3 **Pathogenic**	SCV001149719.1	-	Morinière et al. 2014
**ATS-F533-II-4 (M)**	Germany	Yes	No	AS	7	Yes	Yes	No	Yes	Yes (AS)	24	*COL4A3*	chr2:g.228029470C>T	c.28C>T	p.(Gln10*)	Heterozygous	Father	0.000008252	ARAS	PVS1, PM2 **Likely pathogenic**	SCV001150047.1	-	Novel
												COL4A3	chr2:g.228110696C>A	c.351C>A	p.(Thr117*)	Heterozygous	Unknown	Not listed		PVS1, PM2 **Likely pathogenic**	SCV001150048.1	-	Nagel et al. 2005

**ATS-F687-II-1 (M)**	Serbia	Yes	No	AS	6	Yes	No	No	Yes	No	No	*COL4A3*	chr2:g.228160013_228160015dup	c.3546_3548dup	p.(Gly1183dup)	Heterozygous	Mother	Not listed	ARAS	PS4_moderate, PM1_strong, PM2, PM4_supporting **Likely pathogenic**	-	00375232	Novel
												COL4A3	chr2:g.228172594T>C	c.4421T>C	p.(Leu1474Pro)	Heterozygous	Unknown	0.002664		PS4_moderate (in trans with pathogenic variants), PM1, PM3, PP3 **Likely pathogenic**	SCV001149720.2	-	Chatterjee et al. 2013
**ATS-F688-II-1 (M)**	Serbia	Yes	No	TBMN	17	Yes	Yes	No	Yes	No	No	*COL4A3*	chr2:g.228122337G>T	c.1006G>T	p.(Gly336Cys)	Heterozygous	Unknown	Not listed	TBMN/ADAS#	PS4_moderate, PM1_strong, PM2, PP3 **Likely pathogenic**	-	00375234	Fallerini et al. 2014
**ATS-F257-II-1 (M)**	Macedonia	Yes	No	AS	3	Yes	Yes	No	No	No	No	*COL4A4*	chr2:g.227958838_227958889del	c.1321_1369+3del	p.(?)	Homozygous	Mother and Father	Not listed	ARAS	PVS1_strong, PS4_moderate, PM2 **Likely pathogenic**	SCV001149722.1	-	Novel
**ATS-F503-II-1 (F)**	Croatia	Yes	No	TBMN	4	Yes	No	No	No	No	No	*COL4A4*	chr2:g.(227927315_227942609)_(227945266_227946830)del	Deletion of exons 24 and 25	-	Heterozygous	Unknown	Not listed	TBMN/ADAS#	PVS1_strong, PM2 **Likely pathogenic**	-	00375235	Novel
**ATS-F505-I-1 (M)**	Germany	N.A	No	TBMN	n.d.	Yes	Yes	No	No	No	68	*COL4A4*	chr2:g.227920687C>T	c.2690G>A	p.(Gly897Glu)	Heterozygous	Unknown	Not listed	TBMN/ADAS#	PM1_strong, PM2, PP3 **Likely pathogenic**	-	00375236	Lemmink et al. 1996
**ATS-F641-II-1 (F)**	Macedonia	Yes	No	TBMN	n.d.	Yes	No	No	No	No	No	*COL4A4*	chr2:g.227896735C>T	c.3743G>A	p.(Gly1248Glu)	Heterozygous	Unknown	0.00006053	TBMN/ADAS#	PM1_strong, PM2, PP3 **Likely pathogenic**	-	00375237	Novel
**ATS-F673-II-1 (F)**	Germany	Yes	No	TBMN	10	Yes	No	No	Yes	No	No	*COL4A4*	chr2:g.227942676C>T	c.1921C>T	p.(Arg641*)	Heterozygous	Father	0.000008051	TBMN/ADAS#	PVS1, PM2 **Likely pathogenic**	SCV001430095.1	-	Novel
**ATS-F733-II-1 (F)**	Serbia	No	N.A	TBMN	n.d.	Yes	Yes	No	No	Yes (AS)	No	*COL4A4*	chr2:g.227896691G>A	c.3787C>T	p.(Gln1263*)	Heterozygous	Unknown	Not listed	TBMN/ADAS#	PVS1, PM1, PM2 **Pathogenic**	-	00375238	Novel
**ATS-F7-II-1 (F)**	Germany	Yes	No	AS	34	N.A.	N.A.	No	No	No	N.A.	*COL4A5*	chrX:g.107842023G>A	c.1871G>A	p.(Gly624Asp)	Homozygous	Unknown	0.00008743 (4 hemizygotes)	XLAS	PS4_moderate, PM1_strong, PP3 **Likely pathogenic**	SCV001150062.2	-	Martin et al. 1998
**ATS-F8-I-1 (M)**	Germany	Yes	No	AS	6	N.A.	N.A.	No	Yes	No	N.A.	*COL4A5*	chrX:g.107869530G>A	c.3197G>A	p.(Gly1066Asp)	Hemizygous	Unknown	Not listed	XLAS	PS4_moderate, PM2, PM5_strong, PP3, BP1 **Likely pathogenic**	SCV001149733.1	-	Novel
**ATS-F88-II-1 (F)**	Germany	No	No	AS	22	N.A.	N.A.	No	No	Yes (AS)	N.A.	*COL4A5*	chrX:g.107845121_107845123del	c.2048_2050del	p.(Pro683_Gly684delinsAG)	Heterozygous	*De-novo*	Not listed	XLAS*	PS2, PM2, PM4 **Likely pathogenic**	SCV001150065.1	-	Novel
**ATS-F91-II-1 (F)**	Germany	Yes	No	TBMN	0	N.A.	N.A.	No	No	No	N.A.	*COL4A5*	chrX:g.107842023G>A	c.1871G>A	p.(Gly624Asp)	Heterozygous	Unknown	0.00008743 (4 hemizygotes)	XLAS*	PS4_moderate, PM1_strong, PP3 **Likely pathogenic**	SCV001150062.2	-	Martin et al. 1998
**ATS-F173-II-2 (M)**	Germany	Yes	No	AS	3	N.A.	N.A.	Yes	No	Yes (AS)	N.A.	*COL4A5*	chrX:g.107842023G>A	c.1871G>A	p.(Gly624Asp)	Hemizygous	Mother	0.00008743 (4 hemizygotes)	XLAS	PS4_moderate, PM1_strong, PP3 **Likely pathogenic**	SCV001150062.2	-	Martin et al. 1998
**ATS-F184-II-2 (M)**	Romania	Yes	No	AS	3	N.A.	N.A.	No	No	No	N.A.	*COL4A5*	chrX:g.107850027dup	c.2300dup	p.(Lys768Glnfs*9)	Hemizygous	Mother	Not listed	XLAS	PVS1, PM2 **Likely pathogenic**	SCV001150066.1	-	Novel

**ATS-F236-II-2 (M)**	Spain	Yes	No	AS	7	N.A.	N.A.	Yes	Yes	No	N.A.	*COL4A5*	chrX:g.107844648_107844649del	c.1976_1977del	p.(Ile659Asnfs*11)	Hemizygous	Mother	Not listed	XLAS	PVS1, PM2 **Likely pathogenic**	SCV001150064.1	-	Novel
**ATS-F242-IV-1 (F)**	Macedonia	N.A	No	AS	28	N.A.	N.A.	Yes	Yes	No	N.A.	*COL4A5*	chrX:g.107858210G>T	c.2465G>T	p.(Gly822Val)	Heterozygous	Unknown	Not listed	XLAS*	PM1_strong, PM2, PP3 **Likely pathogenic**	SCV001150070.1	-	Novel
**ATS-F243-II-1 (F)**	Macedonia	N.A	No	TBMN	n.d.	Yes	No	No	No	No	N.A.	*COL4A5*	chrX:g.107842023G>A	c.1871G>A	p.(Gly624Asp)	Heterozygous	Father	0.00008743 (4 hemizygotes)	XLAS*	PS4_moderate, PM1_strong, PP3 **Likely pathogenic**	SCV001150062.2	-	Martin et al. 1998
													chrX:g.107850105G>A	c.2378G>A	p.(Gly793Glu)	Heterozygous	Father	Not listed		PS4_supportin, PM2, PM5, PP3 **Likely pathogenic**	SCV001150069.1	-	Novel
**ATS-F246-II-1 (M)**	Macedonia	No	No	AS	7	N.A.	N.A.	No	Yes	Yes (TBMN)	N.A.	*COL4A5*	chrX:g.107816885dup	c.546+2dup	p.(?)	Hemizygous	Unknown	Not listed	XLAS	PS4_moderate, PM1_strong, PM2, PM4 **Pathogenic**	SCV001149726.1	-	Hashimura et al. 2014
**ATS-F247-II-1 (M)**	Macedonia	No	No	AS	4	N.A.	N.A.	Yes	Yes	Yes (TBMN)	N.A.	*COL4A5*	chrX:g.107838737A>C	c.1424-2A>C	p.(?)	Hemizygous	*De-novo*	Not listed	XLAS	PVS1_strong PS2, PM2 **Pathogenic**	SCV001150058.1	-	Novel
**ATS-F250-II-1 (F)**	Macedonia	N.A	No	TBMN	n.d.	N.A.	N.A.	No	No	Yes (normal)	N.A.	*COL4A5*	chrX:g.107840692G>A	c.1673G>A	p.(Gly558Asp)	Heterozygous	Unknown	Not listed	XLAS*	PS4_supporting, PM1_strong, PM2, PM5, PP3 **Pathogenic**	SCV001150059.1	-	Bekheirnia et al. 2010
**ATS-F260-II-1 (M)**	Serbia	Yes	No	AS	14	N.A.	N.A.	N.A.	N.A.	No	N.A.	*COL4A5*	chrX:g.107935992_107935993ins	c.4543_4544ins	p.(Cys1515Phefs*7)	Hemizygous	Mother	Not listed	XLAS	PVS1, PM2 **Likely pathogenic**	SCV001149740.1	-	Novel
**ATS-F261-II-1 (F)**	Germany	Yes	No	FSGS	5	N.A.	N.A.	No	No	Yes (FSGS)	N.A.	*COL4A5*	chrX:g.107821218G>T	c.645+1G>T	p.(?)	Heterozygous	Unknown	Not listed	XLAS*	PVS1_strong, PM2 **Likely pathogenic**	SCV001149728.1	-	Novel
**ATS-F263-II-1 (F)**	Germany	No	No	AS	10	N.A.	N.A.	Yes	Yes	No	N.A.	*COL4A5*	chrX:g.107802338dup	c.188dup	p.(Phe64Ilefs*16)	Heterozygous	*De-novo*	Not listed	XLAS*	PVS1, PM2 **Likely pathogenic**	SCV001149723.1	-	Novel
**ATS-F267-II-2 (F)**	Kosovo	Yes	No	AS	4	N.A.	N.A.	N.A.	N.A.	No	N.A.	*COL4A5*	chrX:g.(107898688_107908736)_(107979573_?)del	Deletion of exons 38-51	-	Heterozygous	Mother	Not listed	XLAS*	PVS1 PS4_supporting, PM2 **Pathogenic**	-	00375240	Hashimura et al. 2014
**ATS-F274-III-14 (F)**	Germany	No	No	TBMN	32	N.A.	N.A.	No	No	Yes (FSGS, FGGS)	N.A.	*COL4A5*	chrX:g.107850086G>A	c.2359G>A	p.(Gly787Arg)	Heterozygous	*De-novo*	Not listed	XLAS*	PS2 PM1_strong PM2, PM5, PP3 **Pathogenic**	SCV001150068.1	-	Novel
**ATS-F295-II-1 (F)**	Germany	Yes	No	AS	17	N.A.	N.A.	No	No	No	N.A.	*COL4A5*	chrX:g.107821183G>A	c.611G>A	p.(Gly204Asp)	Heterozygous	Mother	Not listed	XLAS*	PS2, PM1_strong, PP3, PM5 **Pathogenic**	SCV001149727.1	-	Gross et al. 2002
**ATS-F336-II-1 (M)**	Russia	Yes	No	AS	4	N.A.	N.A.	No	No	Yes (AS)	N.A.	*COL4A5*	chrX:g.107841951G>T	c.1799G>T	p.(Gly600Val)	Hemizygous	Mother	Not listed	XLAS	PM1_strong PM2, PM5, PP3 **Likely pathogenic**	-	00375241	Novel
**ATS-F486-II-1 (M)**	Germany	Yes	No	AS	3,5	N.A.	N.A.	Yes	Yes	No	N.A.	*COL4A5*	chrX:g.107802377del	c.225del	p.(Gln76Lysfs*79)	Hemizygous	Mother	Not listed	XLAS	PVS1, PM2 **Likely pathogenic**	SCV001149724.1	-	Novel

**ATS-F513-II-1 (M)**	Romania	Yes	No	AS	21	N.A.	N.A.	No	No	No	N.A.	*COL4A5*	chrX:g.107911665G>T	c.3721G>T	p.(Gly1241Cys)	Hemizygous	Mother	Not listed	XLAS	PS4_moderate PM1_strong PM2, PP3<br>**Likely pathogenic**	SCV001149736.1	-	Novel
**ATS-F519-II-2 (F)**	Romania	Yes	No	AS	1	Yes	No	No	No	No	N.A.	*COL4A5*	chrX:g.107929260G>A	c.4217-1G>A	p.(?)	Heterozygous	Unknown	Not listed	XLAS*	PVS1, PM2<br>**Likely pathogenic**	SCV001149738.1	-	Gross et al. 2002
**ATS-F523-II-1 (M)**	Macedonia	Yes	No	TBMN	1	Yes	No	No	No	No	N.A.	*COL4A5*	chrX:g.107863585G>T	c.2606G>T	p.(Gly869Val)	Hemizygous	Mother	Not listed	XLAS	PM1_strong, PM2, PM5<br>**Likely pathogenic**	SCV001149732.1	-	Novel
**ATS-F530-II-1 (F)**	Germany	Yes	No	AS	20	N.A.	N.A.	No	No	No	N.A.	*COL4A5*	chrX:g.107842023G>A	c.1871G>A	p.(Gly624Asp)	Heterozygous	Unknown	0.00008743 (4 hemizygotes)	XLAS*	PS4_moderate, PM1_strong, PP3<br>**Likely pathogenic**	SCV001150062.2	-	Martin et al. 1998
**ATS-F535-II-1 (M)**	Lithuania	Yes	No	AS	22	N.A.	N.A.	Yes	No	No	N.A.	*COL4A5*	chrX:g.107911629G>A	c.3685G>A	p.(Gly1229Ser)	Hemizygous	Mother	Not listed	XLAS	PS4_moderate, PM1_strong, PM2, PM5, PP3<br>**Pathogenic**	SCV001149735.1	-	Hanson et al. 2011
**ATS-F618-II-1 (M)**	Romania	Yes	No	AS	12	N.A.	N.A.	No	Yes	No	N.A.	*COL4A5*	chrX:g.107939570C>T	c.5038C>T	p.(Arg1680*)	Hemizygous	Unknown	Not listed	XLAS	PVS1, PM2<br>**Likely pathogenic**	SCV001149741.1	-	Gross et al. 2002
**ATS-F634-II-1 (M)**	Germany	Yes	No	TBMN	1	Yes	No	No	No	No	N.A.	*COL4A5*	chrX:g.107850041G>A	c.2314G>A	p.(Gly772Ser)	Hemizygous	Unknown	Not listed	XLAS	PM1_strong, PM2, PP3<br>**Likely paathogenic**	SCV001150067.1	-	Novel
**ATS-F636-II-2 (F)**	Germany	Yes	No	TBMN	n.d.	Yes	No	N.A.	N.A.	No	N.A.	*COL4A5*	chrX:g.107911716G>A	c.3772G>A	p.(Gly1258Ser)	Heterozygous	Unknown	Not listed	XLAS*	PM1_strong, PM2, PP3<br>**Likely pathogenic**	SCV001149737.1	-	Novel
**ATS-F662-II-2 (F)**	Macedonia	Yes	No	TBMN	35	N.A.	N.A.	No	No	No	N.A.	*COL4A5*	chrX:g.107834874G>A	c.1423G>A	p.(Gly475Ser)	Heterozygous	Unknown	Not listed	XLAS*	PM1_strong, PM2, PP3<br>**Likely pathogenic**	SCV001150057.1	-	Bekheirnia et al. 2010
**ATS-F664-II-1 (M)**	Serbia	Yes	No	AS	1	N.A.	N.A.	No	No	No	N.A.	*COL4A5*	chrX:g.107826150G>A	c.973G>A	p.(Gly325Arg)	Hemizygous	Unknown	Not listed	XLAS	PS1, PS4_moderate, PM1_strong ,PM2, PP3<br>**Pathogenic**	SCV001149731.1	-	Knebelmann et al. 1992

**ATS-F669-II-1 (M)**	Serbia	Yes	No	AS	6	N.A.	N.A.	No	Yes	No	N.A.	*COL4A5*	chrX:g.107826150G>C	c.973G>C	p.(Gly325Arg)	Hemizygous	Mother	Not listed	XLAS	PS1, PS4_moderate, PM1_strong, PM2, PP3 **Pathogenic**	SCV001150055.1	-	Slajpah et al. 2007
**ATS-F674-II-1 (M)**	Serbia	Yes	No	AS	3	Yes	No	No	No	No	N.A.	*COL4A5*	chrX:g.107842023G>A	c.1871G>A	p.(Gly624Asp)	Hemizygous	Mother	0.00008743 (4 hemizygotes)	XLAS	PS4_moderate, PM1_strong, PP3 **Likely pathogenic**	SCV001150062.2	-	Martin et al. 1998
**ATS-F676-II-1 (F)**	Serbia	Yes	No	AS	7	N.A.	N.A.	No	No	No	N.A.	*COL4A5*	chrX:g.107869539G>T	c.3206G>T	p.(Gly1069Val)	Heterozygous	Unknown	Not listed	XLAS*	PS4_moderate, PM1_strong, PM2, PM5, PP3 **Pathogenic**	SCV001149734.1	-	Bekheirnia et al. 2010
**ATS-F677-II-1 (F)**	Serbia	Yes	No	AS	7	N.A.	N.A.	No	No	No	N.A.	*COL4A5*	chrX:g.107869539G>T	c.3206G>T	p.(Gly1069Val)	Heterozygous	Mother	Not listed	XLAS*	PS4_moderate, PM1_strong, PM2, PM5, PP3 **Pathogenic**	SCV001149734.1	-	Bekheirnia et al. 2010
**ATS-F678-II-1 (M)**	Serbia	Yes	No	AS	4	N.A.	N.A.	No	Yes	Yes (AS)	N.A.	*COL4A5*	chrX:g.107842056G>T	c.1904G>T	p.(Gly635Val)	Hemizygous	Mother	Not listed	XLAS	PS4_moderate, PM1_strong, PM2, PM5, PP3 **Pathogenic**	SCV001150063.1	-	Zhang et al. 2018
**HN-F683-I-2 (F)**	Germany	Yes	No	AS	8	N.A.	N.A.	N.A.	N.A.	No	N.A.	*COL4A5*	chrX:g.107819203G>C	c.609+1G>C	p.(?)	Heterozygous	Unknown	Not listed	XLAS*	PVS1_strong, PM2 **Likely pathogenic**	-	00375242	Novel
**ATS-F686-II-1 (F)**	Serbia	Yes	No	AS	5	N.A.	N.A.	No	No	No	N.A.	*COL4A5*	chrX:g.107815044del	c.446del	p.(Pro149Leufs*6)	Heterozygous	Mother	Not listed	XLAS*	PVS1, PS4_supporting, PM2 **Pathogenic**	SCV001149725.1	-	Novel
**ATS-F694-II-1 (F)**	Bosnia and Herzegovina	Yes	No	AS	2	N.A.	N.A.	N.A.	N.A.	No	N.A.	*COL4A5*	chrX:g.(?_107683074)_(107683437_107782975)del	Deletion of exon 1	p.(?)	Heterozygous	Father	Not listed	XLAS*	PVS1, PS4_supporting, PM2 **Pathogenic**	-	00375243	Renieri et al. 1995
**ATS-F707-II-1 (F)**	Serbia	No	No	AS	2	Yes	Yes	No	No	No	N.A.	*COL4A5*	chrX:g.107930866T>G	c.4470T>G	p.(Tyr1490*)	Heterozygous	Unknown	Not listed	XLAS*	PVS1, PM2 **Likely pathogenic**	SCV001149739.1	-	Novel
**ATS-F737-II-1 (F)**	Germany	No	No	AS	2	N.A	N.A.	No	No	No	N.A.	*COL4A5*	chrX:g.107821345del	c.687del	p.(Gly230Valfs*24)	Heterozygous	Unknown	Not listed	XLAS*	PVS1, PM1_strong, PM2 **Pathogenic**	SCV001149729.1	-	Novel

Supplementary Table 2

Index Case (sex)	Origin	Family history	Consanguinity	Clinical Diagnosis (referring clinician)	Y	Microscopic Hematuria	Proteinuria	Eye anomalies	Hearing impairment	Biopsy	ESKF or preemptive kidney transplantation (age)	Gene	Chromosomal position (hg19)	Nucleotide change	Amino acid change	Zygosity	Inheritance	gnomAD v.2.1.1 MAF/occurrence (CNVs)	ACMG criteria and rating	Accession number in ClinVar	Individual ID in LOVD	Reference
**ATS-F521-II-2 (F)**	Romania	No	No	AS	22	Yes	Yes	No	Yes*	Yes (AS)	23	*COL4A3*	chr2:g.228148573G>T	c.2746+1G>T	p.(?)	Heterozygous	Mother	Not listed	PVS1, PM2<br>**Likely pathogenic**	SCV001149716.1	-	Mencarelli et al. 2015
**ATS-F663-II-1 (M)**	Serbia	Yes	No	AS	6	Yes	Yes	No	No	No	N.A.	*COL4A3*	chr2:g.228160013_228160015dup	c.3546_3548dup	p.(Gly1183dup)	Heterozygous	Father	Not listed	PVS1, PS4_moderate, PM2<br>**Pathogenic**	-	00375232	Novel
**ATS-F787-II-1 (M)**	n.d.	Yes	No	AS	n.d.	N.A.	N.A.	N.A.	N.A.	Yes (AS)	N.A.	*COL4A3*	chr2:g.228112275G>T	c.443G>T	p.(Gly148Val)	Heterozygous	Unknown	0.00001424	PS4_moderate, PM1_strong, PM2, PP3<br>**Likely pathogenic**	-	00375244	Malone et al. 2014
**ATS-F788-II-1 (M)**	n.d.	Yes	No	AS	n.d.	N.A.	N.A.	N.A.	N.A.	No	N.A.	*COL4A3*	chr2:g.228142227G>A	c.2083G>A	p.(Gly695Arg)	Heterozygous	Unknown	0.0001114	PS4_supporting, PM1_strong, PM2, PP3<br>**Likely pathogenic**	-	00375245	Wang et al. 2004

Supplementary Table Legends

Supplementary Table 1. Clinical and genetic characteristics of index cases with either two disease-causing variants in *COL4A3* or *COL4A4* or a hemizygous/heterozygous disease-causing variant in *COL4A5*. All listed variants have been submitted to ClinVar or LOVD. ACMG, American College of Medical Genetics [1-4]; AS, Alport syndrome; ESKF, end-stage kidney failure; F, female; FSGS, focal segmental glomerulosclerosis; H, homozygous; h, heterozygous; Hemi, hemizygous; LOVD, Leiden Open Variant Database; M, male; N.A., not available; p, paternal; TBMN, thin basement membrane nephropathy; VUS, variant of uncertain significance; Y, age at first manifestation (years); *, XLAS female carrier; #, nomenclature currently under discussion.

Supplementary Table 2. Clinical and genetic characteristics of index cases with questionable phenotype. All listed variants have been submitted to ClinVar or LOVD. ACMG, American College of Medical Genetics [1-4]; AS, Alport syndrome; ESKF, end-stage kidney failure; F, female; H, homozygous; h, heterozygous; Hemi, hemizygous; LOVD, Leiden Open Variant Database; M, male; N.A., not available; p, paternal; Y, age at first manifestation (years); *, questionable syndromic phenotype because a sister with no renal phenotype also has hearing impairment.

References

1. Abou Tayoun AN, Pesaran T, DiStefano MT, et al. Recommendations for interpreting the loss of function PVS1 ACMG/AMP variant criterion. Hum Mutat 2018;39:1517-1524.
2. Kearney HM, Thorland EC, Brown KK, Quintero-Rivera F, South ST, Working Group of the American College of Medical Genetics Laboratory Quality Assurance C. American College of Medical Genetics standards and guidelines for interpretation and reporting of postnatal constitutional copy number variants. Genet Med 2011;13:680-685.
3. Richards S, Aziz N, Bale S, et al. Standards and guidelines for the interpretation of sequence variants: a joint consensus recommendation of the American College of Medical Genetics and Genomics and the Association for Molecular Pathology. Genet Med 2015;17:405-424.
4. Ellard S, Baple EL, Berry I, et al. ACGS Best Practice Guidelines for Variant Classification 2019. https://wwwacgsukcom/media/11285/uk-practice-guidelines-for-variant-classification-2019-v1-0-3pdf 2019.
